# Supplementary material for: Structure and mechanism of the alkane-oxidizing enzyme AlkB
Source: Nat Commun. 2023 Apr 17;14:2180. doi: 10.1038/s41467-023-37869-z (PMC10110569; doi:10.1038/s41467-023-37869-z)
Supplement: Supplementary file 1 — Supplementary Information [file 41467_2023_37869_MOESM1_ESM.pdf]

Supplementary Table. 1 Cryo-EM data collection, refinement, and validation statistics of FtAlkB

	FtAlkB-nanobody
**Data collection and processing**	
Magnification	130,000
Voltage (kV)	300
Electron exposure ($e^{-}/Å^2$)	50
Defocus range (μm)	1.6-2.2
Pixel size (Å)	0.95
PDB code	8SBB
EMDB code	40303
Symmetry imposed	C1
Initial particle images (no.)	1,739,854
Final particle images (no.)	107,776
Map resolution (Å)	3.59
FSC threshold	0.143
Map resolution range (Å)	2.2-10.7
**Refinement**	
Model resolution (Å)	3.3
FSC threshold	0.5
Model resolution range (Å)	212.8-3.59
Map sharpening B factor ($Å^2$)	-168.9
Model composition	
Non-hydrogen atoms	4049
Protein residues	499
Ligands	3
B factors ($Å^2$)	
Protein	60.44
Ligand	62.51
R.m.s. deviations	
Bond lengths (Å)	0.016
Bond angles (°)	1.334
Validation	
MolProbity score	2.86
Clashscore	17.50
Poor rotamers (%)	3.99
Ramachandran plot	
Favored (%)	85.05
Allowed (%)	14.95
Disallowed (%)	0

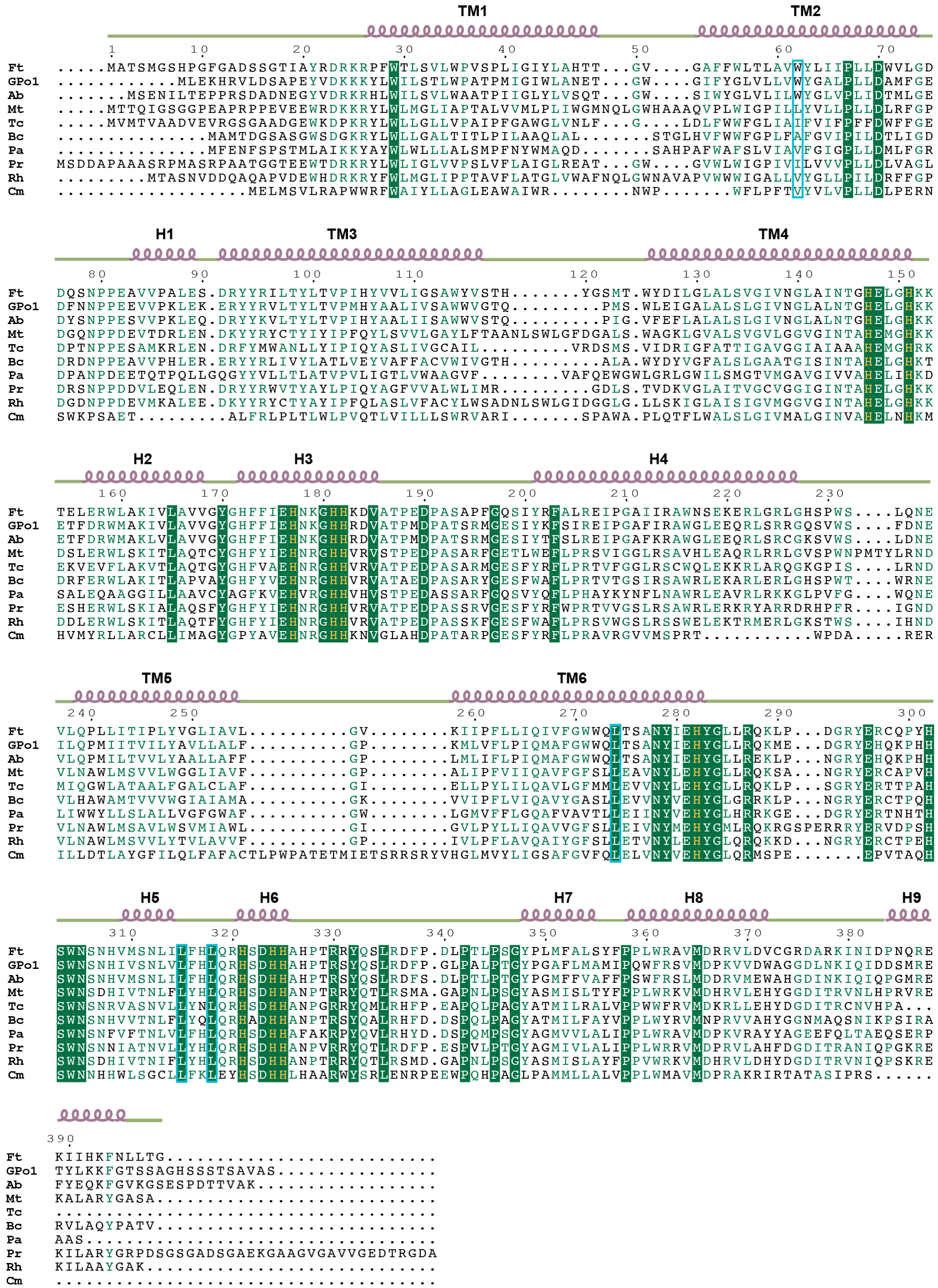

Supplementary Fig. 1 Sequence alignments of AlkBs. Alignments of AlkBs from *Fontimonas thermophila* (Ft), *Pseudomonas putida* GPo1 (GPo1), *Alcanivorax borkumensis* (Ab), *Mycobacterium tuberculosis* (Mt), *Thermomonospora curvata* (Tc), *Burkholderia cepacian* (Bc), *Pseudomonas aeruginosa* (Pa), *Prauserella rugosa* (Pr), *Rhodococcus sp.* Leaf233 (Rh), and *Cyanidioschyzon merolae* (Cm) by Clustal Omega. Sequences are adjusted manually with secondary structures indicated above. Iron coordination residues are highlighted in yellow. Key residues for alkane binding are shown in cyan boxes.

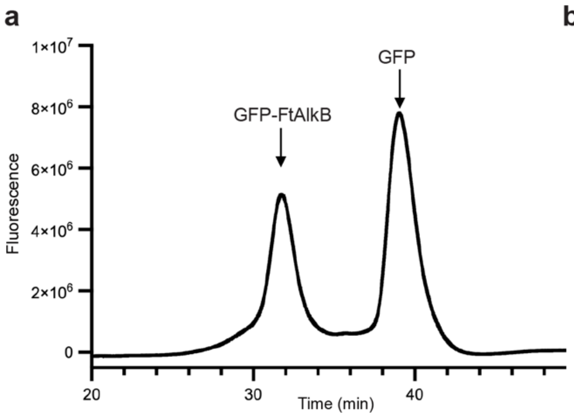

Supplementary Fig.2 Biochemical characterization of FtAlkB. **a,** FSEC profile of GFP-FtAlkB. **b,** A representative size-exclusion chromatography profile of FtAlkB-nanobody complex and the corresponding SDS-PAGE gel. The experiments were repeated independently six times with similar results observed.

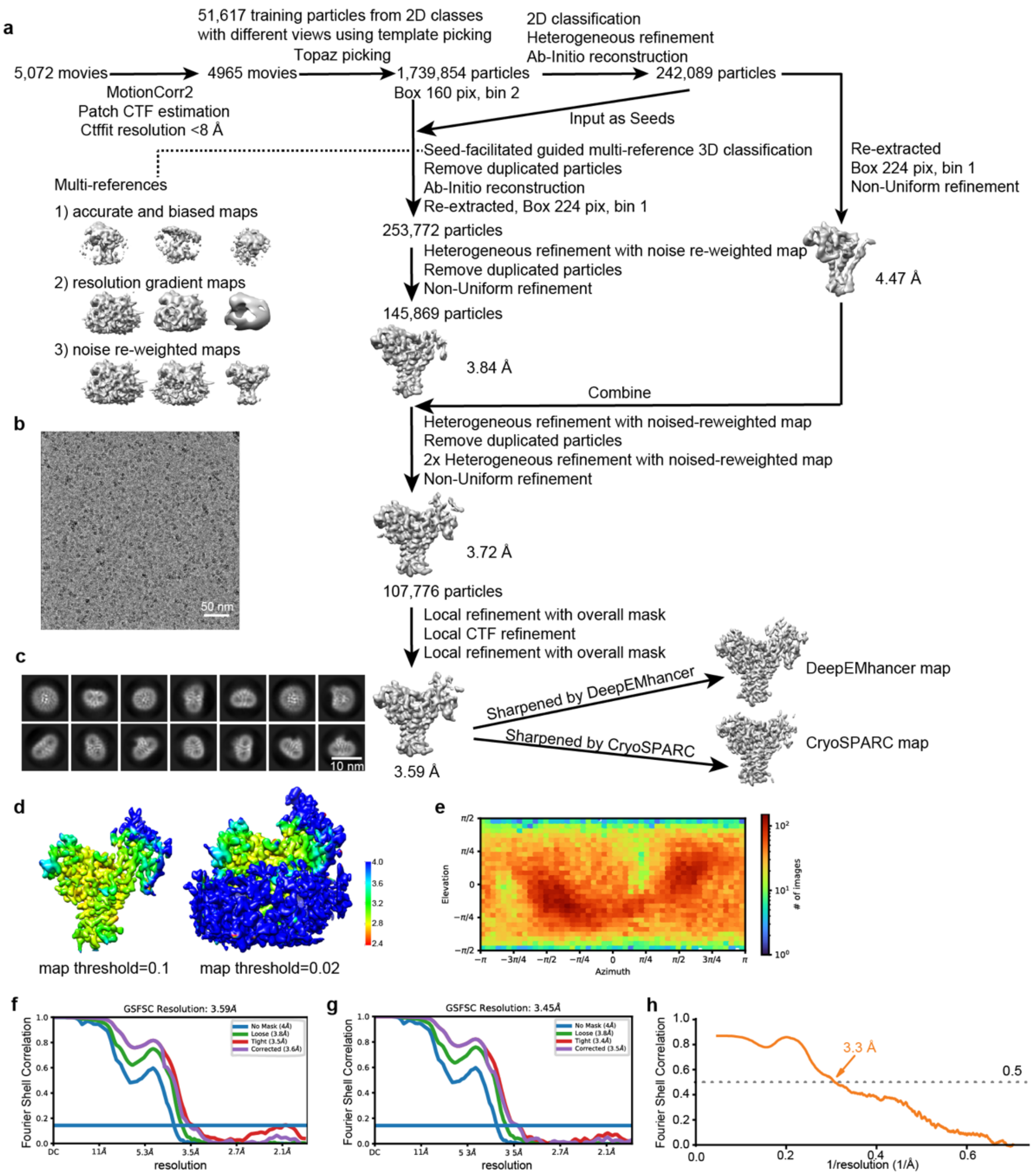

Supplementary Fig. 3 Cryo-EM analysis of FtAlkB. a, Workflow of FtAlkB data processing. **b,** A representative cryo-EM image of FtAlkB-nanobody particles. Details are described in the Methods. **c,** Representative 2D averages. **d,** Local resolution analysis of the FtAlkB-nanobody map. **e,** Angular distribution of particles for the final map. **f,** Gold-standard fourier shell correlation plot of the final FtAlkB-nanobody map. **g,** Gold-standard fourier shell correlation plot of the final FtAlkB map. **h,** Map vs model FSC.

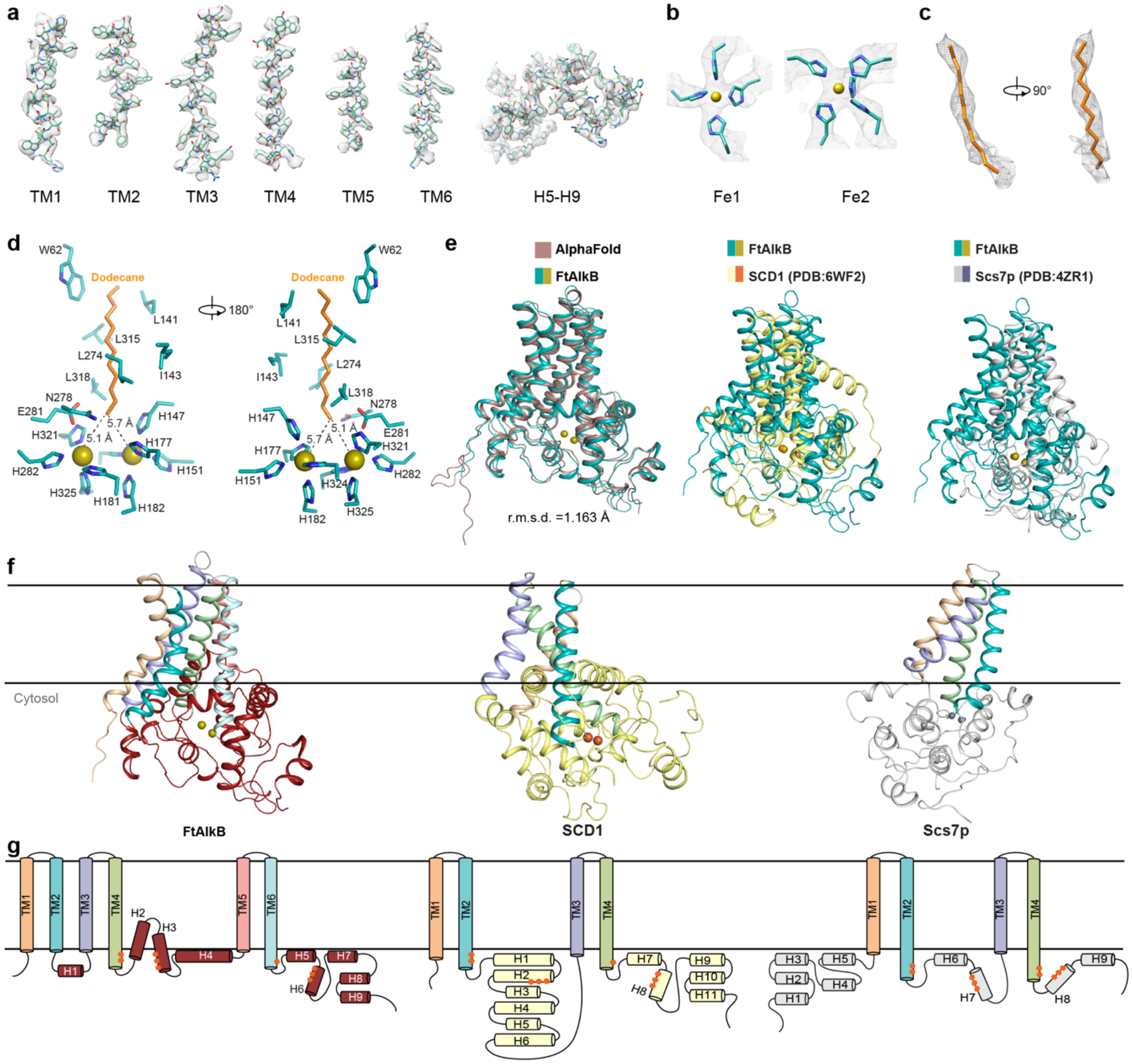

Supplementary Fig. 4 Cryo-EM map of FtAlkB and structural comparison. a, Cryo-EM density maps for all transmembrane helices and the major structure features in the catalytic domain. **b,** Cryo-EM density maps for individual iron and its coordinating histidine residues. **c,** Two views of ligand density fitted with dodecane. Contour level = 8σ. **d,** Two close-up views of the substrate binding channel. Dodecane is shown in orange. Iron ions are shown in sphere. Key cavity residues and iron-coordinating histidine residues are shown in sticks. **e,** Superimpositions of the FtAlkB structure with the AlphaFold model AF-A0A1I2KHB9-F1, SCD1 (PDB: 6WF2), and Scs7p (PDB: 4ZR1). **f-g,** Structures (f) and topology diagrams (g) of FtAlkB, SCD1 and Scs7p. Iron-coordinating histidine residues are shown as orange hexagons.

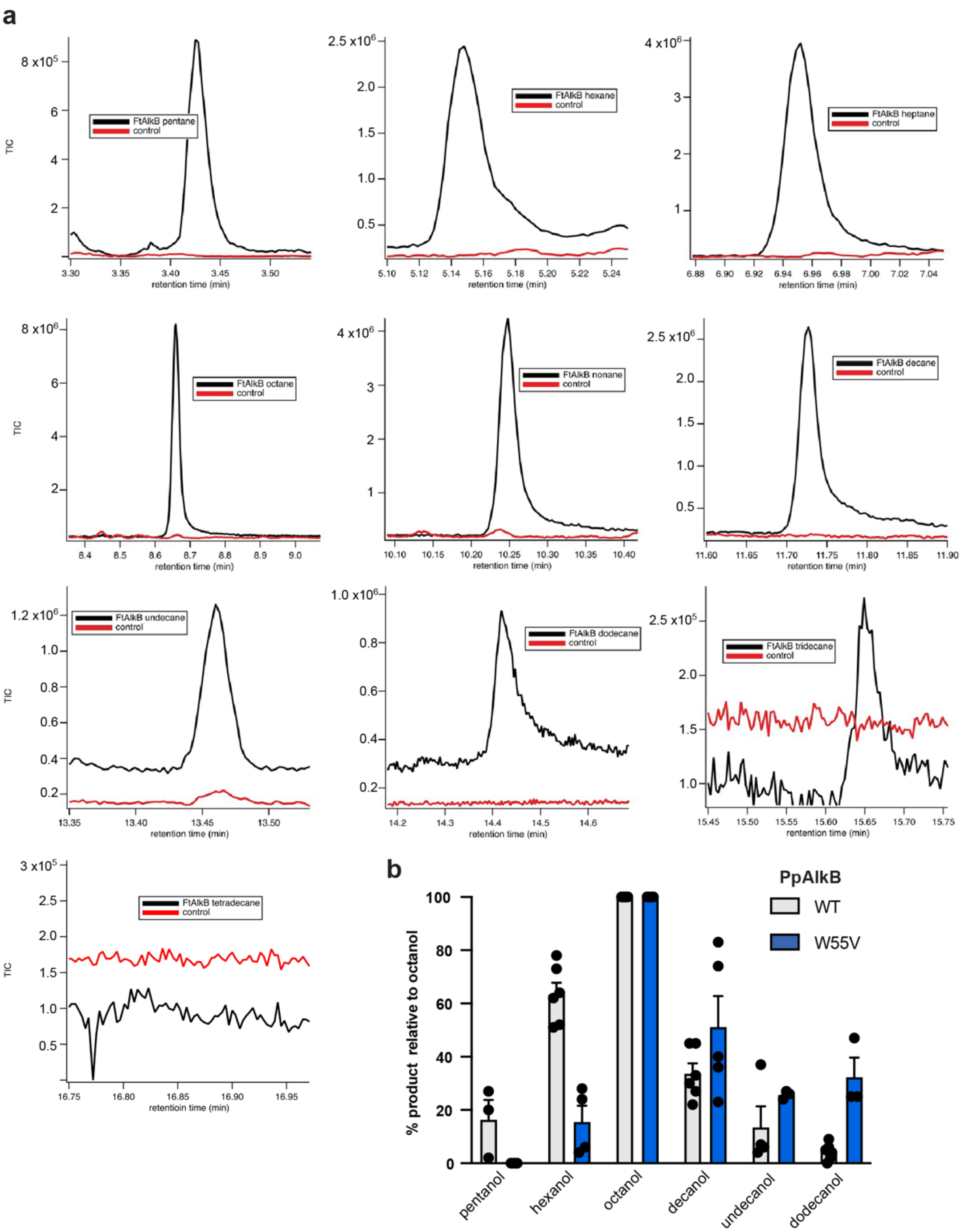

Supplementary Fig.5 Functional characterization of AlkB. **a,** Representing GC-MS profiles of the alcohol peak from the hydroxylation reaction of FtAlkB (black). The negative control traces are shown in red for comparison. Experiments were repeated independently four times and similar results were observed. **b,** Alkane hydroxylation activities of GPo1AlkB WT (grey) and W55V (blue) on straight alkanes (mean ± SEM). From pentane to dodecane, n = 3, 6, 6, 6, 4, 6 independent experiments for WT, n= 4, 4, 5, 5, 3, 3 independent experiments for W55V. Source data are provided as a Source Data file.

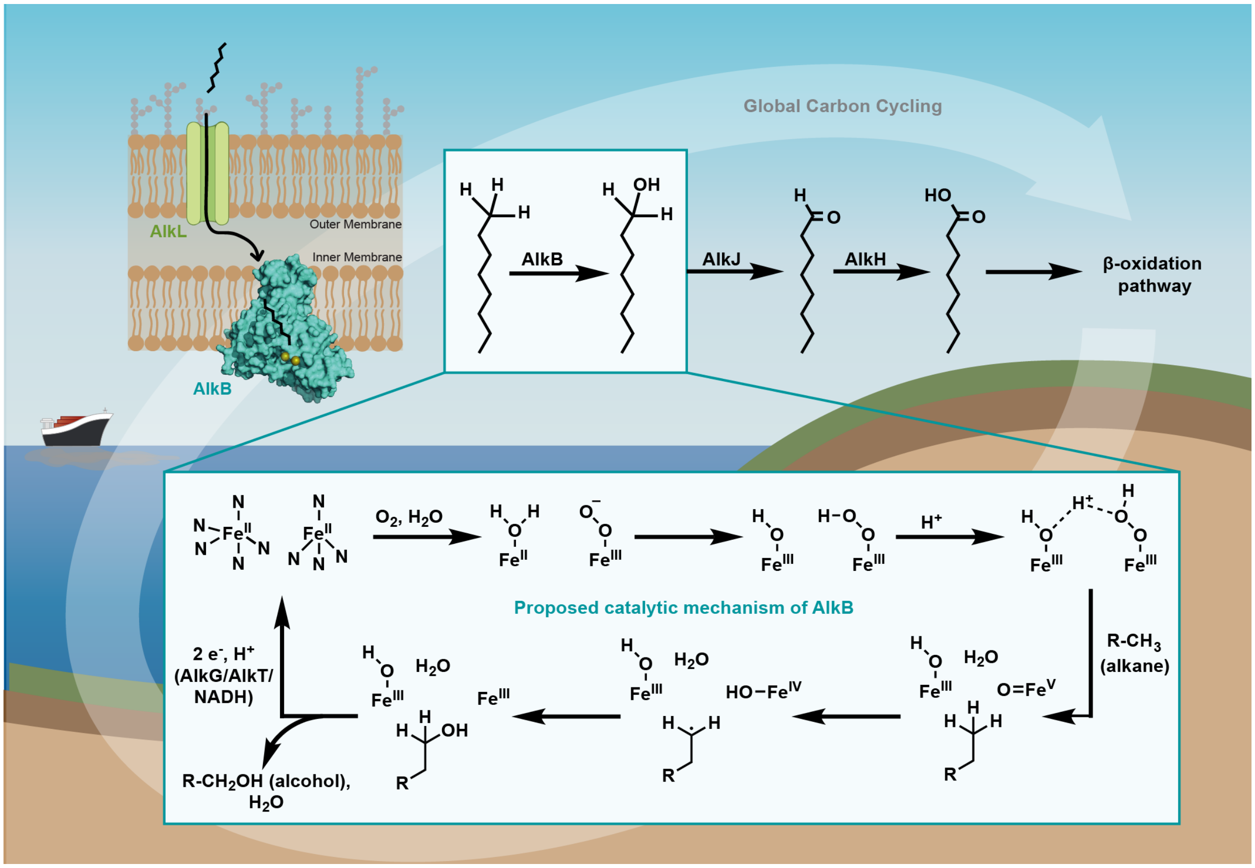

Supplementary Fig.6 AlkB-mediated alkane degradation pathway and proposed catalytic mechanism of AlkB. Microorganisms utilize alkanes as their source of carbon and energy. The transformation of alkanes is initiated by AlkBs, which produce alcohols that are further oxidized before entering the β-oxidation pathway. A catalytic mechanism of AlkB is proposed above based on the long Fe-Fe distance. Other mechanisms are possible, including O-O bond homolysis, which would generate two Fe(IV) species, or a structural rearrangement, not captured in the current structure, that could lead to an sMMO-like mechanism.
